# What makes a reach movement effortful? Physical effort discounting supports common minimization principles in decision making and motor control

**DOI:** 10.1371/journal.pbio.2001323

**Published:** 2017-06-06

**Authors:** Pierre Morel, Philipp Ulbrich, Alexander Gail

**Affiliations:** 1 German Primate Center, Sensorimotor Group, Göttingen, Germany; 2 University of Goettingen, Georg-Elias-Mueller Institute of Psychology, Göttingen, Germany; 3 Bernstein Center for Computational Neuroscience, Göttingen, Germany; University of Oxford, United Kingdom of Great Britain and Northern Ireland

## Abstract

When deciding between alternative options, a rational agent chooses on the basis of the desirability of each outcome, including associated costs. As different options typically result in different actions, the effort associated with each action is an essential cost parameter. How do humans discount physical effort when deciding between movements? We used an action-selection task to characterize how subjective effort depends on the parameters of arm transport movements and controlled for potential confounding factors such as delay discounting and performance. First, by repeatedly asking subjects to choose between 2 arm movements of different amplitudes or durations, performed against different levels of force, we identified parameter combinations that subjects experienced as identical in effort (isoeffort curves). Movements with a long duration were judged more effortful than short-duration movements against the same force, while movement amplitudes did not influence effort. Biomechanics of the movements also affected effort, as movements towards the body midline were preferred to movements away from it. Second, by introducing movement repetitions, we further determined that the cost function for choosing between effortful movements had a quadratic relationship with force, while choices were made on the basis of the logarithm of these costs. Our results show that effort-based action selection during reaching cannot easily be explained by metabolic costs. Instead, force-loaded reaches, a widely occurring natural behavior, imposed an effort cost for decision making similar to cost functions in motor control. Our results thereby support the idea that motor control and economic choice are governed by partly overlapping optimization principles.

## Author summary

Economic choice in humans and animals can be understood as a weighing of benefits (e.g., reward) against costs (e.g., effort, delay, risk), leading to a preference for the behavioral option with highest expected utility. The costs of the action associated with a choice can thereby affect its utility: for equivalent benefits, an action that requires less physical effort will be preferred to a more effortful one. Here, we characterized how human subjects assess physical effort when choosing between arm movements. We show that the effort cost of a movement increases with its duration and with the square of the force it is performed against but not with the distance covered. Therefore, the subjective cost that determines decisions does not reflect the objective energetic cost of the actions—i.e., the corresponding metabolic expenditure. Instead, the subjective cost has commonalities with the cost that our central nervous system is believed to minimize for controlling the motor execution of actions. Our findings thus argue in favor of action selection and action control sharing common underlying optimization principles.

## Introduction

Should I rather bring the groceries from the car trunk to the kitchen in 1 trip or in 2 trips? Even in a seemingly simple decision like this, multiple decision parameters are at odds. When doing a single trip, this bothersome task will certainly be finished more quickly but will require an intense physical effort. This choice might also put one at risk to drop everything on the way. On the other hand, when making 2 trips, each will be less effortful and safer but the task will take longer to complete. When examined through the prism of economics, this example shows 2 alternatives with an equal reward but different amounts and types of costs: risk, time, and effort. Utility theory [1] posits that these decision parameters are combined in a single value, the utility, which characterizes the desirability of each choice as whole.

The ways in which costs affect the utility of an option have been well described for risk (prospect theory [2]) and delay (hyperbolic temporal discounting [3]). Defining such a relationship is not straightforward for effort. Physical effort [4], in contrast to mental effort [5], can at least be related to an external, physically measurable variable, in the same way that reward delay is used in the example of temporal discounting. Therefore, we focus on physical effort, defined here as the subjective cost or negative utility associated with a given motor action, independent from the costs resulting from its success rate (risk) or delayed reward (temporal reward discounting).

Studies focusing on the brain circuits involved in physical effort-based decision making in humans have used handle-squeezing tasks to produce different effort levels, but they just assumed that subjective effort increases monotonically with isometric squeezing force, without further characterizing the dependency [6,7]. Using a similar task, Hartmann and colleagues [8] showed a quadratic discounting of monetary rewards by squeezing force, suggesting that effort for isometric force production grows proportionally to the square of the force amount. In contrast, by pitting isometric force production with different parameters directly against each other, Körding and colleagues defined effort as a function of both the duration and magnitude of force production, without the need to use an external monetary scale [9]. By using 2 parameters in a force-production task, this latter study highlighted the multifaceted nature of physical effort. The use of isometric force-production tasks to probe physical effort discounting is, however, still limiting compared to the full range of effort-related parameters one could experience when deciding between actual movements. Here we characterize the influence of duration, distance, direction, and force on subjective effort costs in actual reaching movements.

From the perspective of motor control, planning and executing a movement, even towards an unambiguous goal, requires commitment to a specific motor act among an infinite amount of potential ways (“choices”) to acquire the goal. Decision making in this context can be seen as part of a continuum that includes motor planning and motor control, and minimizing various cost functions is a core concept of motor control: the stereotypical nature of movement trajectories and velocity profiles has been attributed to minimization of hand jerk [10], endpoint variance [11], and even control effort itself [12,13]. The potential tight link between decision making and motor control is supported by studies showing that action selection can take into account parameters that are related to movement execution, such as biomechanics [14,15] or motor accuracy [16]. Conversely, the vigor with which a movement is executed was shown to be explainable through delay discounting [17]. This raises the question of whether the subjective cost of an action as computed in a decision-making context (i.e., what we call effort here) is comparable to potential cost functions used for optimization in motor control or, as an alternative, to the metabolic cost of the movement.

Here we address this question by investigating how humans assess subjective physical effort in action-selection tasks involving binary choices between different reaching movements. In a first experiment, we varied movement duration, amplitude, and direction as well as resistive force in order to derive isoeffort curves in this duration–amplitude–direction–force space. This allowed us to independently test the sensitivity of subjects to impulse (force × duration) and work (force × amplitude) exerted during movements. In a second experiment, we pitted repeated identical movements against single movements with different resistive forces in order to obtain more precise estimates of the relationship between force and subjective effort in reaching movements.

## Results

### Effects of amplitude, duration, and force on reach effort

**Fig 1:**
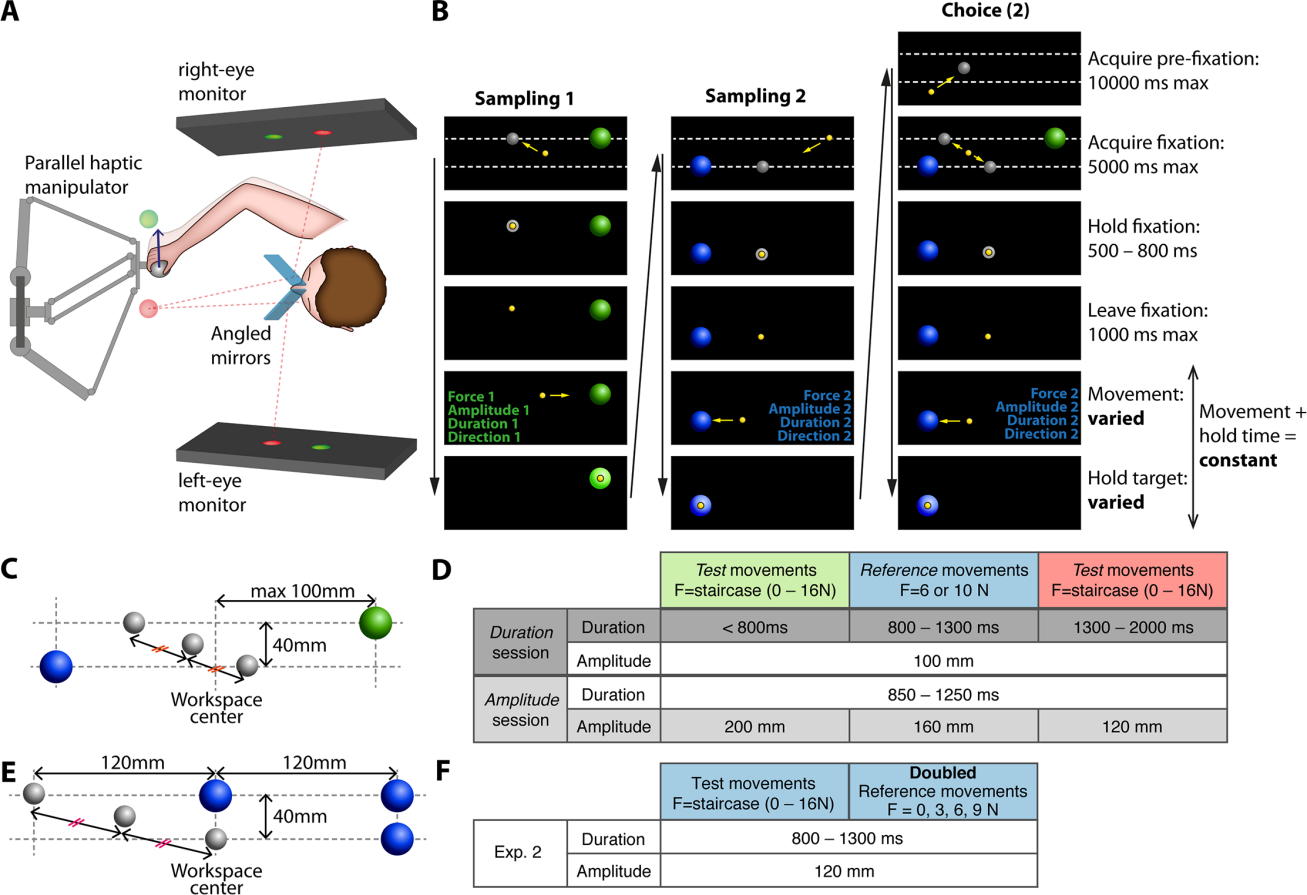
Task design for assessing the effort of reaching. A. Haptic 3D augmented reality setup. Subjects moved a haptic manipulator handle with adjustable resistive force when reaching. Visual stimuli, here pictured as transparent spheres, were stereoscopic virtual images superimposed to the manipulator workspace, seen by the subject through the mirror-based virtual reality display. The dashed red lines represent the virtual image paths for the red sphere. B. Sequence of a single trial in experiment 1 (amplitude session). Each column corresponds to a subtrial (test movement sampling, reference movement sampling, choice) and each row to a different stage during the subtrial (black arrows indicate time progression). The yellow sphere is the cursor coinciding with the manipulator handle location, grey spheres are movement starting points, and blue and green spheres are movement targets. Yellow arrows (representing cursor movement) and the dashed lines (indicating the vertical offset between the 2 alternative movements) are shown for the purpose of illustration. The 2 movements differed in the resistive force generated by the haptic manipulator, in direction, and in either amplitude or duration, depending on the session. In the choice subtrial, the subjects were asked to choose the less effortful movement experienced during sampling by moving to the corresponding starting point in the “acquire fixation” stage (second row) and repeating this movement (movement 2 is chosen in this example). C. Example target locations in experiment 1 (amplitude session). The targets for the 2 alternative movements (green and blue spheres) were equidistant to the workspace center (the maximum distance to center was 100 mm). Horizontal offsets in the position of the starting points (upper and lower grey spheres) created the different movement amplitudes. In the choice subtrials, the middle grey sphere was used as an initial starting point: subjects then indicated their choices by going to the starting point corresponding to the movement they picked. D. Movement constraints in the amplitude and the duration session for experiment 1. Amplitudes correspond to the distance between the centers of the fixation sphere and the target sphere. Colors in the table header correspond to the actual target colors in the corresponding movements, which cued subjects on movement speed. In each trial, a test movement was pitted against a reference movement. E. Example target locations in experiment 2. Trials from experiment 2 were similar to experiment 1, except that the reference action consisted of 2 identical movement repetitions. The upper spheres (grey sphere and 2 blue spheres) represent the starting point and targets of the repeated movement. The 2 lower spheres represent the starting point and target of the single movement. As in experiment 1, subject indicated their choice by going from the middle grey sphere to the starting point of the chosen action (upper or lower grey spheres) F. Movement constraints in experiment 2.

In both experiments, subjects performed 2-alternative forced choice (2-AFC) tasks in which they compared 2 different actions and were asked to choose the least effortful one (Fig 1). To make informed choices in each trial, subjects first performed both proposed actions (sampling) and then reported the least effortful action by executing it again (choice). The need to repeat the chosen action rendered the choice relevant for the subjects, since genuine selection minimized the overall task effort. Both actions consisted of reach movements performed against different levels of resistive force. In each trial, one of the proposed actions served as a reference action, while the other served as a test action. Note that this distinction was not indicated and not relevant to the subjects but was part of our adaptive experimental design. Within each task condition, the trial-to-trial resistive force level in the test movements was selected with a staircase algorithm [18], while the force level of the reference movements was kept constant. As a consequence, the staircases converged to the force level at which the test action was perceived as being equally effortful as the reference action (equivalent force).

In experiment 1, reference and test actions consisted of single movements, differing primarily in amplitude or duration. More precisely, in each trial of the amplitude session, subjects had to choose between 2 movements that differed in amplitude, direction, and force (after sampling both). Conversely, in each trial of the duration session, the choice was between movements that differed in duration, direction, and force. In both sessions, the staircase algorithm adjusted the forces of 1 of the movements, depending on the choice of the subjects, until both movements were subjectively equivalent in effort for the subject. This allowed us to construct isoeffort curves in the force–amplitude–duration movement parameter space (Fig 1A–1D and Methods).

In experiment 2, the reference action consisted of 2 identical repeated movements and the test action of a single movement. This allowed us to determine the scaling of subjective effort with force (Fig 1E and 1F).

### Experiment 1: Subjects prefer short-duration movements independently of amplitude

**Fig 2:**
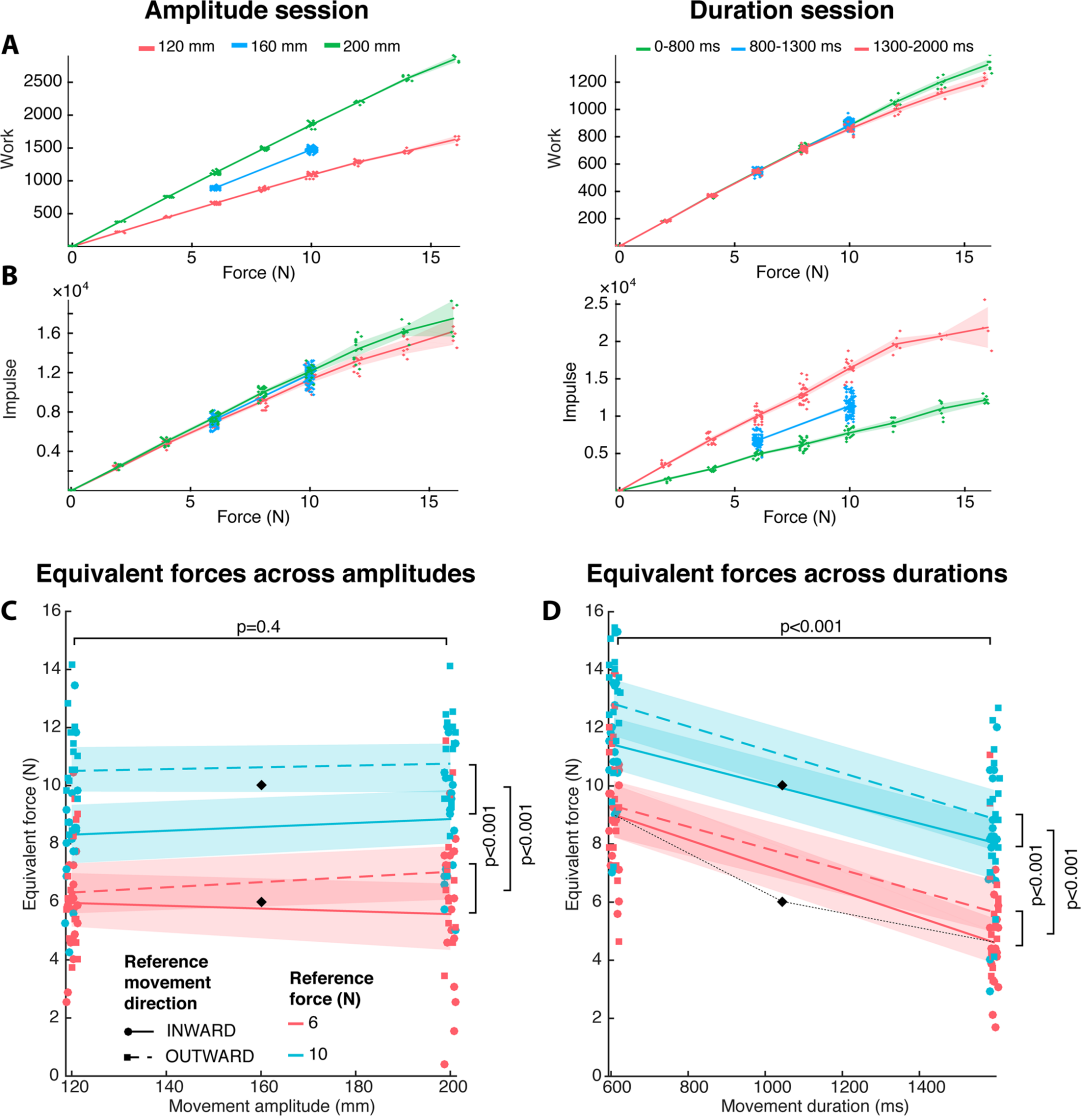
Effort depends on reach duration but not amplitude (results of experiment 1). A. Additional physical work imposed by the manipulator as a function of force level for all reach amplitudes (left) and all reach durations (right) for a representative subject (LM). The shaded areas represent the 95% confidence interval of the mean. Each data point corresponds to the work in 1 subtrial (discrete force levels; points are jittered graphically along the x-axis for better visibility). B. Additional impulse imposed by the manipulator as a function of force for the same subject. C. Equivalent forces (force levels considered by the subjects as equivalent in effort to the reference movement) in the amplitude session of experiment 1. Equivalent forces were computed as the asymptotic forces onto which the adaptive staircase procedures converged (average of staircase inversions). Each colored point represents the equivalent force for a single subject and a single movement condition, and black diamonds represent the reference movements. Data are separated by reference movement force level (color) and direction relative to handedness (symbol and line style). The lines and shaded areas correspond to the mean and 95% CI of the equivalent force across subjects (individual subject’s data are graphically jittered along the x-axis for better visibility). D. Equivalent forces in the duration session of experiment 1. The locations of the points on the x-axis correspond to across-subject averages of movement durations. Otherwise, the same conventions hold as in C. Empirical isoeffort curves can be visualized by connecting each point representing a reference movement (black diamonds) to the corresponding 2 points representing the obtained equivalent movements. For illustration, this panel shows a single example of an empirical isoeffort curve in the force–duration plane, represented as a thin black dotted line. Data underlying this figure can be found at https://doi.org/10.6084/m9.figshare.4873055.v1

In experiment 1, we asked subjects to conduct naturalistic reach movements against different force levels, either with varied durations independent of amplitude (duration session) or with varied amplitudes independent of duration (amplitude session). As we used constant force profiles, these constraints correspond to dissociations either in impulse (force integrated over time) or work (force integrated over distance), respectively. Fig 2A and 2B depict the average work and impulse produced by the manipulator as a function of force for the different duration and amplitude conditions in both sessions for a representative subject (both integrated from the time of movement onset minus 100 ms to movement offset plus 400 ms). Impulse values were well separated in the duration session but not in the amplitude session, while work values were well separated in the amplitude session but not in the duration session. This confirms that visual instructions about reach-target location and requested movement duration together with the manipulator-controlled resistive force successfully constrained the actual movements of the subjects to the desired parameter ranges in each session (sample trajectories and generated force profiles in S1 Fig and S1 Text). Importantly, the forces imposed by the manipulator had an additive effect on the torques that the subject’s arm actually needed to produce to generate the movements. Since the imposed forces were independent from arm kinematics, a simple biomechanical model of the arm (S3 Text, S3 Fig, S4 Fig) showed that over the duration of a movement, the torques the subjects produced to compensate the imposed forces outweighed the torques produced to compensate for the inertia of the arm. As a consequence, the total work and impulse actually produced by the subjects in the different conditions showed dissociations comparable to those observed in Fig 2A and 2B.

Subjects’ choices did not systematically depend on performance differences in the various task conditions of experiment 1, but they depended reliably on force levels (S2 Fig, S2 Text). As a consequence, we could use equivalent forces to titrate the effort subjects associated with the explored movement parameters.

In the amplitude session of experiment 1, the equivalent forces did not vary significantly with movement amplitude (linear mixed-effect model [LME], *p* = 0.4, effect size 0.3 N) but varied significantly with reference force level (LME, *p* < 0.001, effect size 3.4 N) (Fig 2C). The result indicates that the 4-N difference between the reference forces was large enough for subjects to judge it as different in effort but that subjects were unaffected in choice by movement amplitude over the tested range when movement duration was kept constant. In other words, a movement of 120 mm against a force of 6 N was rated as effortful by the subjects as a movement of 200 mm against a force of 6 N. Since movement duration was constant in the amplitude session, the observed insensitivity to movement amplitude can also be interpreted as insensitivity to movement speed.

In contrast, subjects were sensitive to movement duration when movement amplitude was kept constant in the duration session (Fig 2D). Equivalent force levels were lower for long-duration movements than short-duration movements (LME, *p* < 0.001, effect size 3.8 N). Here, a movement in the 1,300–2,000 ms range against a force of 5 N was judged as effortful as a movement in the 0–800 ms range against a force of 9 N. This indicates that long-lasting reaches were perceived more effortful than brief reaches against the same force level. Additionally, the equivalent forces scaled with the reference force levels (*p* < 0.001, effect size 3.1 N) without interaction between the factors force and duration (*p* = 0.4).

Physical movement parameters like work or impulse, as defined above, describe the movement properties at the manipulator handle (endpoint movement), irrespective of the required multijoint arm movement. Instead, effort evaluation and the resulting choice were based on subjective experience, to which the biomechanics of the movement could have contributed. In fact, biomechanics of the movement influenced effort judgment in our experiment. In both the amplitude and the duration sessions of experiment 1, equivalent forces depended on the reference movement direction (Fig 2C and 2D). Test movements performed inward (i.e., towards the left for right-handed subjects and vice versa) required higher force levels to be judged as equally effortful as outward movements (LME, amplitude session: *p* < 0.001, effect size 1.5 N; duration session: *p* < 0.001, effect size 0.9 N). This indicates that at the same force level, outward movements were considered more effortful than inward movements. This difference in equivalent force is likely linked to the use of larger muscles and the higher available strength for inward movements.

After showing that duration and biomechanics but not amplitude had an influence on the effort judgment, we asked how effort would depend on the force itself. In Fig 2D, a thin dotted line represents an example isoeffort curve in the force–duration space: it connects the point representing parameters of a reference movement to the points representing parameters of equivalent-force test movements. Similar to this example curve, when averaging over movement directions in the duration session, isoeffort curves are convex for both reference force levels. This indicates that the putative effort cost function supporting the subjects’ choices was a nonlinear combination of force and movement duration. This is because if effort was a linear combination of force *F* and duration *d*, such as *E*(*F*,*d*) = *αF* + *βd*, the isoeffort curve defining the equivalent force of the test movement would have to be a straight line defined as $F_{T}=\frac{\alpha F_{R}+\beta d_{R}}{\alpha }-\frac{\beta }{\alpha }d_{T}$. If, on the other hand, effort was a purely multiplicative function of force and duration—i.e., assuming that the effort cost function is impulse *E*(*F*,*d*) = *Fd*—then this would lead to convex isoeffort curves shaped as the inverse function $F_{T}=\frac{F_{R}d_{R}}{d_{T}}$. The isoeffort curve in Fig 2D is not straight, and the curvature does not fit the impulse model but is shallower instead. In experiment 2, we determined the precise shape of the force–effort relationship for constant movement durations.

### Experiment 2: The cost function for effort is proportional to squared force

Binary choices between 2 options only allow ranking the options in terms of preferred or nonpreferred. To describe effort as a function of force, additional information is needed to turn such a ranking into a scale with a continuous metric. This could be achieved by trying to compensate the effort cost of an action with an independent scalable benefit (e.g., monetary reward) to achieve equal preference for movements of different force. But the utility function of the benefit must then be known, a task which might be as difficult to achieve as the task of determining the effort cost function itself. As an alternative, in each trial of experiment 2, subjects chose between 2 options after having sampled both: either they opted for performing 2 similar movements in rapid succession against a reference force level *F*_*R*_ (the endpoint of the first movement is the starting point of the second movement), or they chose to perform a single movement against a test force level *F*_*T*_ (adjusted between trials by a staircase). Here we assume that an action consisting of 2 identical movements is twice as effortful as an action consisting of 1 of these movements, but both give the same benefit (finishing the trial). Under this assumption, the equivalent forces *F*_*Teq*_ (staircase convergence point for forces *F*_*T*_ in single test movements) as a function of the reference movement force level *F*_*R*_ (fixed forces in double movements) should follow the rule *E*(*F*_*Teq*_) = 2*E*(*F*_*R*_), that is *F*_*Teq*_ = *E*^−1^(2*E*(*F*_*R*_)), with *E*(*F*) being the function linking force and subjective effort (= cost function) and *E*^−1^ being its inverse. As in experiment 1, the observed decision behavior in experiment 2 was best explained by force-based choices rather than performance-based choices (S2 Text). Results from experiment 2 thus allowed to test and fit models for both the cost function and its link to decisions, which we carried out using a Bayesian modeling approach (see Methods).

**Fig 3:**
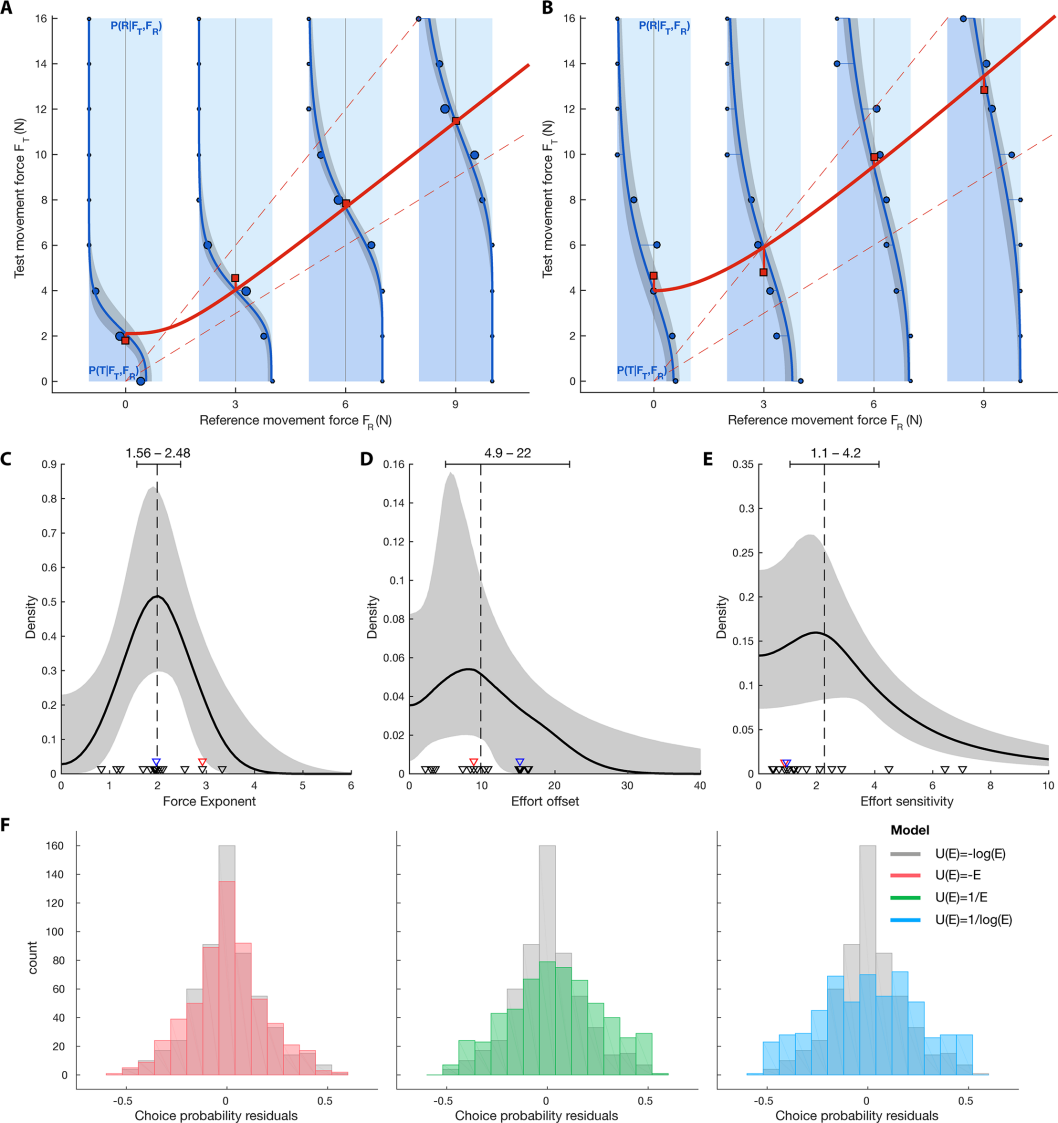
Effort depends on squared movement force (results of experiment 2). The Bayesian fit of data from experiment 2 using Eq 1 and Eq 2 revealed a force exponent of 2 for linking effort and force. A and B. Subject-level fits of the choice behavior for subjects JP and MK. The fitted equivalent force curve (thick red line, Eq 3) represents the estimated test movement force *F*_*Teq*_ at which the test movement felt as effortful as the corresponding double reference movement. The underlying psychometric functions in blue illustrate how this functional relationship is linked to the subject’s choice behavior. For each of the sampled reference movement forces *F*_*R*_ (0, 3, 6, and 9 N, major x-axis), the estimated choice probability *P*(*R*|*F*_*T*_,*F*_*R*_) of choosing the reference movement over the test movement is plotted as function of test-movement force in the vertical plots (blue line). For a given *F*_*T*_ (i.e., along the corresponding horizontal line), the width of the dark blue area represents the probability for the subject to pick the test movement, and the width of the light blue area represents the probability for the subject to pick the double reference movement. The darker region corresponds to the 95% credible interval extracted from the posterior samples. Note that the fitted equivalent force curve intersects the choice probability curves at their points of subjective equality (i.e., verifies *P*(*R*|*F*_*Teq*_,*F*_*R*_) = 0.5). The blue discs represent the subjects’ actual choice probabilities (disc areas are proportional to the number of trials. Equivalent forces computed as in experiment 1 are shown as red squares for comparison to the Bayesian fit. For reference, the unity line and *F*_*Teq*_ = 2*F*_*R*_ line are depicted with dotted red lines. C. Representation of the population-level compound posterior distribution for the force exponent *α*. This compound distribution represents the distribution of *α*’s posterior distributions, constructed from the sampled posterior distributions for *α*’s population mean and variance. The black line and the grey area represent the median of the compound distribution and its 95% credible interval. The dashed line and corresponding error bar indicate the median and 95% credible interval for the posterior of the population mean of the force exponent *μ*_*α*_. Triangles represent the medians of the subject-level posteriors for the force exponent *α*_*i*_ (red: subject shown in A; blue: subject in B). The value of 2 for the force exponent describes a squared force dependency of effort. D. Population-level compound posterior distribution for the effort offset. E. Population-level compound posterior distribution for the effort sensitivity *γ*. F. Distribution of residuals for the choice probability curves. The selected model (in grey, Eq 4) is compared to the alternative models (other colors). The residuals correspond to the signed distances between the blue curves and the blue discs on panels A and B. Residuals from all subjects are represented here. Data underlying this figure can be found at https://doi.org/10.6084/m9.figshare.4873055.v1

A direct observation of the equivalent force as a function of the reference force level is suited to highlight the properties required of *E*(*F*). We computed equivalent forces in 2 ways: first as averages of the test-force levels at the staircase inversions (i.e., the asymptotic force to which the staircase procedure converged as in experiment 1) and second via points of subjective equality of the psychometric functions that resulted from the Bayesian model. Results of both approaches are illustrated for 2 example subjects in Fig 3A and 3B. Equivalent to the considerations regarding the isoeffort curves in experiment 1, the simplest putative effort cost function is a linear function, *E*(*F*) = *αF*. For this, equivalent forces would have to obey the equation *F*_*Teq*_ = 2*F*_*R*_ (steepest dashed red line), as we required *E*(*F*_*Teq*_) = 2*E*(*F*_*R*_). However, in our data we observed that for large reference forces, the equivalent forces were smaller than predicted from the linear model. For example, for a *F*_*R*_ of 9 N, subjects JP and MK showed equivalent forces of 12 and 14 N, respectively, instead of 18 N. This observation indicates that there was a convex nonlinear relationship between force and effort, in line with the results obtained from experiment 1. Additionally, we observed that the equivalent forces for a *F*_*R*_ of 0 N were larger than 0 N, confirming the intuition that a movement against no external force still has nonzero effort (i.e., that E(0) > 0). Therefore, a power function with an offset, (*F*) = *F*^*α*^ + *β*, appears as a reasonable minimal model for the force–effort relationship (Eq 2; see Methods), which we will use in the following sections.

Our Bayesian modeling approach used the trial-by-trial choices of subjects as dependent data. Thereby, within the same unified framework, we simultaneously modeled (1) how forces affected effort values (see paragraph above, Eq 2) and (2) how the subjects’ choices depended on these effort values (Eq 1). For the dependence of choice on effort, we answered 2 questions. Does utility show a subtractive discounting by effort (i.e., the difference in utilities on which decisions are based is equivalent to a difference between efforts in our task [Eqs 4 and 5]) or a hyperbolic discounting by effort (i.e., the difference in utilities corresponds to a difference between effort inverses [Eqs 6 and 7]). Within each of these 2 alternatives, we tested whether effort was represented in a linear (Eqs 5 and 6) or logarithmic scale (Eqs 4 and 7). This 2 x 2 design resulted in 4 alternative models obeying *E*(*F*_*Teq*_) = 2*E*(*F*_*R*_), all with the same number of free parameters (see Methods). Notably, all tested models had the same equation for the equivalent force curve (Eq 3); the models only varied in the shapes of the choice curves—in particular, how the reference force *F*_*R*_ affected their slope. We assessed model quality on the basis of 3 criteria. First, the percentage of correctly predicted choices (binary predictions based on a comparison between the actual test force and fitted equivalent-force levels) reflected the validity of the equivalent force curve as a decision boundary (in red on Fig 3A and 3B). Second, we evaluated the fit of the model to the full choice probability curves (in blue on Fig 3A and 3B) by examining the corresponding residual distribution (Fig 3F). Last, each fit was also tested by using the Watanabe–Akaike Information Criterion (WAIC) [19], an approximation of cross-validation that allowed us to compute the relative likelihood between models. The model we ultimately selected (and show in Fig 3A and 3B) expressed choice probability as a function of the difference between the logarithms of reference and test efforts (Eqs 1, 2 and 4) and outperformed all other models, which we discuss below.

The simplest model obeying *E*(*F*_*Teq*_) = 2*E*(*F*_*R*_) was based on a difference between test and reference efforts (Eq 5, no logarithms). The difference model predicted subjects’ choices as well (68.3% correct predictions) as the selected logarithmic difference model (68.7%), meaning that the equivalent force curves were similar. But the difference model did not fit the choice probability curves nearly as well (WAIC of 4.50e3) as the selected model (4.43e3), making the difference model less likely by a factor of 6.3e–16 compared to the selected logarithmic difference model. Indeed, the distribution of residuals for the simplest model (in red, Fig 3F), is wider than the distribution of residuals for the selected model (in grey, Fig 3F). Both alternative models based on hyperbolic discounting of effort, whether computing choice probability from the difference between the inverse of efforts (Eq 6) or the inverse of effort logarithms (Eq 7), showed lower prediction performances (66.9% and 63.8%), higher WAICs (4.76e3 and 5.09e3), and wider residual distributions than the selected model and therefore had to be rejected.

The results of this model-selection approach thus favor a subtractive discounting of utility by effort (no hyperbolic discounting) and a logarithmic internal representation of effort. By examining the posterior distributions of the selected model’s parameters (Fig 3C–3E, Eqs 1 and 2), we can interpret the equivalent force curves obtained in our subject population. Our main parameter of interest was the population average of the force exponent *α* in the power function described in Eq 2, as it describes the nonlinear dependency of effort on force. The exponent *α* showed a narrow posterior distribution centered around 2. This means that on average, the subjective effort rose with the square of the resistive force against a movement. Since the distribution is narrow, the confidence in this estimate is high (95% credible interval for the population average: 1.56–2.48). For completeness, the posterior for the effort offset *β* (95% CI: 4.9–22), reflecting the effort that subjects associated with performing a movement against no resistive force, and the posterior for *γ* (95% CI: 1.1–4.2), reflecting the sensitivity of subjects to effort differences, are represented in Fig 3D and 3E, respectively.

The biomechanics seemed to have played less of a role in experiment 2. For the same reference force levels, we did not obtain different equivalent test forces between the 2 movement directions inward and outward. However, in contrast to experiment 1, subjects never directly compared movements with different directions, as both options were in the same direction. This difference likely made experiment 2 much less sensitive in that respect.

## Discussion

In this study, we used an action-selection task to characterize how physical effort discounts the utility associated with arm movements and controlled for potential confounding factors such as delay discounting and performance. By repeatedly asking subjects to choose between 2 arm movements of different amplitude or duration that were performed against different levels of force, we were able to construct isoeffort curves in the amplitude–duration–force parameter space. These isoeffort curves indicated that for a choice between 2 arm movements against resistive forces, the movement amplitude did not influence effort cost but its force and duration did: movements with a longer duration were judged more effortful than shorter movements against the same force. Biomechanics of the movements also influenced their utility, as movements towards the midline of the body were less effortful than movements away from it. In a second experiment, by using the same approach but introducing movement repetitions as factor, we determined that the cost function in effort-based decisions had a quadratic relationship with force, and that choices were made on the basis of the logarithm of these cost functions.

### Influence of force on effort in movements compared to isometric contractions

Most studies on physical effort in human decision making operationalize effort by asking subjects to squeeze a handle that measures hand grip force [7,8,20,21], a device that is easy to use and fMRI-friendly. The effort is then an isometric contraction of varying magnitude, expressed as a percentage of the maximum voluntary contraction (%MVC) that each subject can produce. Subjects are typically required to choose between 2 squeezes with different grip forces, each associated with different rewards or additional decision factors (delay or risk). Hartmann and colleagues associated monetary rewards with grip forces and reported that among linear, hyperbolic, and quadratic effort cost functions, the quadratic cost function explained the subjects’ behavior best [8]. Klein-Flügge and colleagues used a similar task to compare effort discounting and delay discounting and reported that effort cost seemed best described as a sigmoidal function, i.e., showed a convex dependency for lower forces, as Hartmann and colleagues did, but Klein-Flügge and colleagues found a concave relationship for forces closer to MVC because of saturation of effort cost [20]. Burke and colleagues, instead, compared the integration of physical effort and risk in a similar task and reported a sharp increase of effort cost when approaching MVC [21]. In contrast, Prevost and colleagues used rewarding erotic images instead of money, but found effort cost to fit a hyperbolic function [7]. This means that for isometric force production, the effort cost function is still uncertain or at least depends on the choice task (reward, risk, or delay discounting), while for actual movements hardly any previous data exist.

To answer how effort depends on force in our movement task, we first have to address the question of how choice is best linked to effort, since choice is the behavioral readout, while effort is a hidden decision variable. Apart from the study by Prevost and colleagues, the aforementioned studies rejected the idea that choice behavior takes into account physical effort in a similar fashion to delay (i.e., by hyperbolic discounting). This is not surprising since, intuitively, high physical effort cannot make the subjective utility of a choice decrease asymptotically to zero (as hyperbolic discounting does because of the increasing denominator). Indeed, the subjective utility of a high-effort, low-reward action could well be negative, in which case doing nothing would be preferable. As a consequence, the cost of effort is a value that should be offset from the benefits of an action in the utility space—i.e., the utility of each action should be computed by subtracting the associated effort. This intuition is confirmed by the results of our experiment 2: models in which the decision variable was a difference between efforts predicted the subject’s movement choices better than models in which the decision variable was a difference between the inverse of efforts. Effort studies with isometric force production modeled the probability of choosing each option by transforming the difference of efforts between the 2 alternatives with the softmax function [7,20]. Here we used an equivalent probit transformation but showed that using the difference of the effort logarithms yielded significantly better results than using the difference of efforts. Indeed, the difference of logarithmic effort as choice variable best captured the decrease in sensitivity we observed for higher efforts (Fig 3A and 3B).

How does force affect effort in transport movements? Previous isometric contraction studies do not allow generating a good prediction for this question. The aforementioned studies modeled the subjective cost of effort as convex functions of force expressed as %MVC and titrated efforts against rewards. However, both these features of the experimental design could overemphasize the convexity of the effort cost function. First, producing a force stronger than MVC is by definition impossible. Therefore, the effort cost likely has to undergo a sharp increase when approaching this discontinuity in designs that use the full 0%–100% range of MVC as a force scale. Indeed, Hartmann and colleagues and Burke and colleagues noted that subjects always chose the effortful option when it provided more reward, except when the effort was close to MVC [8,21]. Second, the tendency to almost always make reward-based choices while ignoring moderate efforts also suggests that monetary rewards are too strongly motivating for typical subjects and may not be appropriate to study the cost of the moderate efforts. However, moderate efforts are essential in experimental settings to allow large numbers of trials and to stay away from the MVC discontinuity. In conclusion, a paradigm that uses moderate absolute forces instead of %MVC forces and in which effortful actions are not associated with monetary or social rewards but are compared directly seems more suited to precisely determine effort cost functions.

Such a paradigm, which we partly adopted here, was introduced for isometric forces by Körding and colleagues in a study in which subjects had to resist against imposed force profiles of variable magnitudes and durations [9]. This previous study led to a different effort cost function than the one we found. When subjects had to choose between dual and single contractions with the same force profiles, but in which force and duration were varied together, a loss function of the form (*FT*)^*α*^ gave the best fit for = 1.1. Assuming that this fit can be generalized to constant durations, the resulting *F*^1.1^ relationship would indicate a quasilinear influence of isometric force on subjective effort. In contrast, in our experiment 2, in which we extended this approach to actual effortful transport movements and isolated force dependency by keeping duration constant, we found a more convex *F*^2^ relationship. Hence, our result is closer to the results obtained in studies using %MVC despite the use of a different task, force scale, and fitting procedure (the force exponent was a free parameter in our model, in contrast with [8]). Nevertheless, our use of moderate forces prevents us from generalizing our findings to movements realized against higher levels of forces (closer to MVC), for which large accuracy and duration changes might bias choice preferences independent of force-dependent effort.

In summary, the cost of effort as a function of isometric muscle contraction force has previously been shown to take various forms. Yet, we mainly attribute differences to the quadratic discounting that we observed here to 2 facts that were not fully considered previously. First, compensating effort with rewards is difficult because of uncertainties about the reward utility itself and the need to use large forces. Second, even when avoiding reward–effort competition and comparing effortful actions directly, interactions between force and duration need to be avoided or compensated for, since duration contributes to both effort and reward discounting and thereby may distort measured force–effort relationships, as will be discussed in the following paragraph.

### Influence of duration on effort in movements compared to isometric contractions

To properly understand physical effort in movements, it is important to disentangle the different contributions of force and duration. A previous study obtained V-shaped isoeffort curves in the duration–force space when pitting isometric contractions with force profiles of different durations and magnitudes against each other [9]. For durations below 250 ms, an increase of duration required a sharp decrease of force to maintain effort constant; for durations above 500 ms, the opposite was observed. In other words, contractions of long durations (1–2 s) were considered as effortful as shorter contractions, even with slightly lower forces. This observation contradicts the intuition that longer contractions should be more effortful than shorter contractions. Moreover, performing short contractions allowed finishing the experiment more quickly since total trial duration was not controlled for; therefore, delay discounting should have additionally devalued longer movements. Körding and colleagues interpreted their result as a consequence of increased control difficulty for fast force changes: it was easier for subjects to resist against stronger force profiles when the onset and offset of the forces were slower, which was the case for long-duration force profiles. In this sense, their results marked a compound effect. In contrast, in our task we tied the onset and offset of forces to self-timed movements. This rendered force control less difficult, and, as a consequence, we observed monotonically decreasing isoeffort curves in the duration-force space (Fig 2D)—i.e., effort increased monotonically with both duration and force, as intuitively expected.

Other recent work argued that increasing movement durations requires smaller and smaller decreases of force to maintain effort constant (i.e., that perceived effort reaches an asymptote instead of growing linearly with duration in isometric force productions). To explain such a counterintuitive effect, the authors assume that effort costs are subject to the same temporal discounting as can typically be observed for reward in economic choice behavior [22]. This hypothetical explanation is, however, not applicable to our results, as we kept the total duration of trials constant. The isoeffort curves observed in our experiment 1 can be explained by the quadratic relationship between force and effort.

In conclusion, for effortful transport movements like reaches, effort increases monotonically with movement duration, suggesting that effort is integrated over time.

### Movement cost in motor control and decision making

The observations from experiment 1 and 2 provide insight into the internal cost function used by subjects to decide between arm movements. This effort cost function for decisions could potentially be paralleled with motor control cost functions or with the actual metabolic cost of movements. In other words, we can examine whether the choice made by subjects between proposed movements with constrained parameters (duration, speed, force, etc.) reflect natural preferences in the execution of unconstrained movements or minimization of energy expenditure.

#### Control cost

Invariants in movement kinematics have been interpreted as evidence for the minimization of cost functions during motor control (control costs). While earlier work concentrated on cost functions directly related to movement kinematics or dynamics, such as movement jerk [10], the field turned towards more behaviorally relevant cost functions, such as endpoint variance [11] or a combination of error and integrated squared control signal (“control effort” in [13]). The similarity between these behaviorally relevant cost functions for motor control and putative costs for decision making led to the formulation of powerful unified frameworks in which control cost is balanced with movement duration and movement accuracy [17,23,24]. Here we tested the link between motor control and economic choice by evaluating whether control cost can explain the choice behavior in our subjects.

With our experimental design, we were able to isolate control cost as a potential decision cost, since we controlled for temporal reward discounting with waiting periods that compensated the different movement durations and since we used large target sizes and forgiving movement termination parameters to prevent variations in movement accuracy from influencing performance and hence choice. We observed an increase in effort with movement duration in experiment 1 and a quadratic dependency of effort cost on force in experiment 2, which is consistent with the integrated squared control signal proposed in the motor control literature (control signals are low-pass filtered to generate forces [13]). These results suggest that effort costs used in decisions and motor control are similar and thus might result from overlapping minimization principles.

This notion might appear at odds with some of the results of experiment 1 when considering the actual forces the subjects produced. Indeed, subjects needed to counter both the force applied by the manipulator and the inertia of their arm. While the resistive forces we used were constant, inertial forces increased with movement speed. As a consequence, one could expect effort to increase for high-speed movements, but this was not the case in either session. This apparent contradiction was resolved by building a simple model of the arm to estimate the forces generated by the subjects. Indeed, when integrated over the whole movement duration, the torque necessary to compensate the constant force applied on the arm’s distal extremity dominated the torque generated by the subjects (S4C and S4D Fig: movement speed had a negligible effect on actual impulse). This could explain a reduced sensitivity to movement speed without violating the assumption that minimizing control costs explains the choice behavior.

#### Energetic cost

Energetic cost of movements has also been proposed as a putative cost for motor control [25–27] but does not appear to be suitable for the cost function used by subjects in our experiment. Muscular energetic costs are expressed as energy rates, implying a linear increase of energy consumption with time, and depend on both force and speed of contraction. The increase of energy rates with force in nonisometric contractions appears to be close to linear, while energy rates show a nonmonotonic relationship with speed of muscle shortening [28]. When considering whole movements and not single muscles, the energy rate is linearly related to the squared speed for walking [29]. Reaching shows similar relationships in which, at constant durations, the energy expended for movements depends almost linearly (exponent = 1.1) on distance [22]. The use of energetic cost as a decision factor is thus neither consistent with the lacking amplitude dependency in our experiment 1 nor with the quadratic force–effort relationship observed in experiment 2.

Last, we observed that for the same force and duration, subjects preferred movements toward the body midline to movements away from the body midline. This result confirms that subjects take into account biomechanical properties of effectors in decisions [14,15]. This preference is likely not explained by differences in metabolic costs. We used diametrically opposing movement directions with identical inertial properties of the arm, as theoretically expected from symmetry considerations and empirically supported by the fact that movement peak acceleration and velocity (depending on reach direction) form ellipses [22,30]. Rather, direction preference here seems linked to higher strength availability and the use of larger muscles for inward movements. Indeed, the inward movements involved elbow flexion and shoulder adduction, both of which show higher maximal strength than their opposite contractions [31,32]. The dependence of choices on available muscle strength could thus also be linked to control costs. Indeed, it has been proposed that control costs are inversely proportional to muscle strength, as stronger muscles show less motor noise [33].

In summary, by taking into account squared force and available muscle strength, the subject's decisions were more consistent with the use of control costs rather than metabolic costs as decision variables. The match with control costs could appear partial, as decisions were not affected by distance and/or speed in our experiment; however, this was likely due to the kinematic-independent resistive forces we used. Additionally, we purposefully decoupled distance and duration, whereas in natural conditions, both are tightly linked; therefore, movement duration might be an effective shortcut used to determine effort cost in general cases. Last, we isolated effort as a cost in our experiment, but effort can also be rewarding in some contexts (exercising, sports competitions), in which case the utility can increase with effort.

## Methods

After providing informed written consent, 17 subjects participated in experiment 1, and 16 other subjects participated in experiment 2 (ages 19–30 years, 6 left-handed subjects, normal or corrected-to-normal vision, no overlap in subjects between the experiments). Experiments were in accordance with institutional guidelines for experiments with humans, adhered to the principles of the Declaration of Helsinki, and were approved by the ethics committee of the Georg-Elias-Mueller-Institute for Psychology at the University of Goettingen. In both experiments, subjects answered a postexperiment questionnaire.

### Augmented-reality haptic reach setup

Subjects performed the tasks by holding and moving the spherical handle of a parallel-type haptic manipulator (Delta.3, Force Dimension, Nyon, Switzerland) with their dominant arm (Fig 1A). The manipulator was connected to a computer running our own custom-written software (C++, OpenGL) in charge of visual stimulus presentation, task event control, force computation, and associated data recording. The manipulator and the computer communicated bidirectionally at 2 kHz, with the manipulator sending the 3D position of the handle and the computer requesting forces to be applied at the handle for each iteration of this 0.5-ms haptic cycle.

The movements of the manipulator handle were reproduced in real time for the subject via a spherical yellow cursor displayed in a stereoscopic augmented-reality (3D-AR) environment. Display and haptic device latency were fully compensated by a forward prediction to achieve synchrony between visual cursor and handle movement (Kalman filter with position, speed, and acceleration as state variables). The 3D-AR environment consisted of 2 computer monitors (BenQ XL2720T, screen size 590 x 338 mm, 60-Hz refresh rate, distance 45 cm, Matrox DualHead2Go DisplayPort splitter) that were placed to either side of the subject with the screens facing each other. The subject viewed the screens through a pair of semitransparent mirrors that were angled at 45° relative to the screens. This allowed for the creation of stereoscopic 3D visual stimuli that were perceived as being projected into the haptic device’s workspace. In addition to the visual cursor, which always coincided with the handle's current physical position, other visual stimuli indicated the starting points and targets of the reaching movements as well as text information.

The 3D-AR haptic interface was calibrated for each subject. For this, we made the actual manipulator handle visually coincide with multiple visual targets sequentially presented in the virtual space. Since the control software selected the visual target locations, the manually adjusted handle position could be used to compute the manipulator-to-display transformation matrix for the current geometry of the setup. This calibration was then further adjusted for each subject by setting the location and projection matrix of the virtual openGL cameras according to the subject’s interpupillary distance.

To allow the subjects to comfortably operate the haptic manipulator, both monitors and mirrors were tilted to lower the location of the 3D representation (Fig 1A; angle relative to horizontal: 30°). For the same reason, we defined a virtual plane in front of and parallel to the monitor image plane in which all movement targets appeared (distance to mirrors was 430 mm). Subjects were also encouraged to take breaks and relax their arm as frequently as desired. In order to limit the force output of the manipulator to task-relevant forces only, and to allow for natural movement trajectories, subjects could freely move the cursor around the entire spherical workspace of the haptic device. Correct depth perception of the 3D stimuli was thus required for the subjects to be able to acquire the movement targets.

Before each experimental session, subjects were trained on simple versions of the tasks in order to familiarize themselves with the setup and the task requirements, notably 3D vision, resistive forces, and time constraints.

### Force generation

The haptic manipulator produced forces that resisted the subjects' movements. Our aim was to produce a force with constant magnitude that was only present during the movements and that opposed the instantaneous movement direction, similar to a kinetic friction force. The direct definition of this friction force would thus depend on the velocity of the handle. Yet, the force command sent to the manipulator was not computed directly from online estimates of handle velocity (which is difficult at low speeds) to prevent sudden force onset and direction inaccuracies at low handle speeds. Instead, we implemented the friction force using a virtual point-mass (virtual mass = 100 g) that was connected to the handle via a virtual spring (coefficient = 1 N.m^-1^). In other words, subjects dragged a virtual mass with the help of a spring, and the kinetic friction force was computed according to the speed of the virtual mass and applied to it. The magnitude of this kinetic friction force (in N) was varied in order to produce the different resistive force levels. For each iteration of the haptic cycle, the dynamic state of this virtual mass was updated according to the forces applied to it (= sum of the spring force and the friction force), and the force resulting from the virtual spring was sent to the haptic manipulator as a command. The position of the virtual point-mass was reset to the handle location, and thereby the spring force set to 0, before the start of each movement. Additionally, the commands sent to the haptic manipulator were modulated by an envelope function (a constant function with linear tapers of varied durations at onset and offsets), which allowed controlling force output outside of the defined movement periods.

### Experiment 1

The course of events of a trial in experiment 1 is presented in Fig 1B (example trial from the amplitude session). Each sampling subtrial started with the subject placing the cursor (6-mm diameter, yellow sphere) within a fixation sphere (20-mm diameter, grey, brightening upon acquisition) and holding this position for a duration randomized between 500 ms and 800 ms. The movement target sphere (diameter 30 mm) was displayed from the start of the subtrial; its color indicated to the subject the modalities of the movement to be executed: across both sessions, a green target indicated the need for a rapid movement (a short-duration movement in the duration session or a large-amplitude movement in the amplitude session), whereas blue and red indicated medium and low speed, respectively. During the acquisition and hold stages, onscreen text announced which sampling movement was currently being performed (“sampling 1” or “sampling 2”). When the fixation sphere disappeared (“go” cue), the subject had to execute the required movement by placing the cursor within the target sphere within the requested time constraints. Movement duration was computed from movement onset (determined online by a combination of speed and distance thresholds) to target acquisition (determined only based on cursor position relative to the target). Resistive forces were turned on when the fixation sphere was acquired (on-taper: linear increase to the desired force value within 200 ms) and were turned off when the target was acquired (off-taper: linear decrease to 0 within 600 or 500 ms in case of successful or failed acquisition). Note that the actual force production by the manipulator was dependent on the subject’s movement and only started at movement onset (see "Force generation" section). If the subject acquired the target faster than the minimum duration set for the movement, or if the subject did not reach the target before the maximum duration set for the movement, the subtrial was interrupted and onscreen text indicated to the subject the type of error committed (“too fast” or “too slow”). Failed subtrials, which also included trials in which the subject broke fixation or left the target too early, were restarted until executed correctly. Once the target was acquired (with movement duration *d*_*m*_), which was signaled by the target sphere becoming brighter, the subject had to hold the cursor within the sphere for a total duration (in ms) *d*_*h*_ = 100 + *d*_*mmax*_ − *d*_*m*_, with *d*_*mmax*_ being the maximum potential movement duration in the session (2,000 ms in the duration session, 1,250 ms in the amplitude session). This ensured that every subtrial had the same duration across conditions within a session, thus preventing temporal discounting, here equivalent to the desire to terminate the experiment early by preferably selecting short movements.

After sampling both the test movement and the reference movement by performing each sampling subtrial successfully, the subject had to indicate in the choice subtrial which movement felt less effortful. This subtrial, announced by a “choice” onscreen text, started with the subject acquiring a pre-fixation sphere. Then, the fixation spheres and targets for the 2 alternatives were displayed, and the subject indicated their choice by acquiring the fixation sphere of the chosen movement (Fig 1B, right column). With acquisition of the chosen movement’s fixation sphere, the fixation and target spheres of the nonchosen movement disappeared, and the rest of the subtrial was identical to the sampling subtrial that corresponded to the chosen movement.

Subjects performed experiment 1 over 2 sessions on different days, with each session lasting on average 70 minutes. In the duration session, reference and test movements differed in allowed movement duration but had the same amplitude. This allowed us to construct isoeffort curves in the force–duration space. Conversely, in the amplitude session, the reference and test movements differed in amplitude, but not in duration, which allowed us to construct isoeffort curves in the force–amplitude space. With the use of constant magnitude force profiles, the duration and amplitude session allowed us to double-dissociate total impulse ($J_{x}=\int_{t_{start}}^{t_{stop}}F_{x}dt$) and work ($W_{x}=\int_{t_{start}}^{t_{stop}}F_{x}\frac{dx}{dt}dt$), respectively. The reference movements had a medium duration in the duration session (800–1,300 ms) and medium amplitude in the amplitude session (160 mm). In both sessions, reference movements were performed against either 6 N or 10 N of resistive force and could be directed either to the right or to the left. The test movements required either low or high values for the session variable of interest (low or high duration or amplitude) and were carried out in the direction opposite to the reference movements (Fig 1D). These combinations lead to 8 conditions per session, which were presented to the subject in a randomly interleaved manner (2 reference movement force levels × 2 reference movement directions × 2 test movement levels of duration or amplitude).

For each of these 8 conditions, independent pairs of staircases determined the force level against which the test movements were performed. These staircase pairs followed a one-up one-down rule with a step size of 2 N, with one staircase starting at 0 N and the other at 16 N (the highest force the manipulator could sustainably produce) to compensate hysteresis. In other words, when the subject chose the reference movement in a given trial, the force level of the test movement in the next trial of the same condition and staircase would be decremented by 2 N; and vice-versa, when the subject chose the test movement, the force level of the next test movement of the same condition and staircase was incremented by 2 N. Data collection for each staircase was considered complete after 7 inversions in the subject’s choices. In this subjective-choice task, the choices of the subject could sometimes lead the staircase procedure to propose force values beyond the capabilities of the manipulator (above 16 N) or the interest of the task (below 0 N). As a consequence, the force values were clamped between 0 and 16 N, and each time the force stayed at these boundaries for 2 trials in a row, an inversion was counted in order to allow the staircase to terminate eventually. We used the average force at staircase inversions to determine isoeffort forces in experiment 1. Because of the clamping of the staircase forces, the isoeffort forces were also bounded between 0 and 16 N, which could have caused an underestimation of the observed effects in the rare cases in which the subject stayed at the clamped force limits.

Target and fixation locations were selected to avoid confounding biases. Across all 8 conditions, the targets were not placed further than 100 mm from the workspace vertical midline to prevent the effort of reaching towards large eccentricities, which would be considered a confounding factor. To achieve this, the different movement amplitudes in the amplitude session were created by offsetting the locations of the fixation spheres while keeping the targets at constant eccentricities (Fig 1C, vertical dotted lines). For this reason, the 2 alternative movements (towards left and right) also had to be placed at different heights on the workspace (40-mm vertical distance). This made them visually more distinguishable for the subjects, especially in the choice subtrial, in which both targets and both corresponding fixation spheres are displayed. Importantly, the prefixation sphere in the choice subtrial was placed halfway between the 2 alternative fixation spheres to prevent subjects from choosing the movement starting closest to the current cursor location. Both fixation spheres were visually identical and were identified by their vertical position, which was the same as the target of the corresponding movement.

### Experiment 2

While experiment 1 was designed to explore isoeffort curves in the force–duration–amplitude spaces, the similar experiment 2 was designed to provide more details about the shape of the force–effort relationship. Instead of executing a single movement in the reference action, subjects performed an identical movement twice in experiment 2, while the amplitudes and durations of all movements were kept the same across conditions. Assuming that executing a movement twice doubles the associated effort, the experiment allowed us to determine how much force in a test movement was needed to double the effort of a single movement from the reference action. Subjects performed experiment 2 in a single session (average duration 140 minutes).

To repeat the reference movement, 2 targets were presented in the corresponding subtrial, and subjects performed 2 reaches in succession. The location of the first target was used as a starting point for the second movement such that no additional movements were required (additional movements would cause more than doubling of effort). Targets were placed such that the 2 movements matched in reach direction and amplitude (Fig 1E). In a reference action, after the movement to the first target, the subject had to maintain the cursor in its location for 500 ms, after which the first target disappeared, indicating to the subject to perform the movement to the second target. The resistive force was tapered in and out for each of these movements (50-ms onset taper on “go” cue and 400-ms offset taper on target acquisition). In all other aspects, the course of events for experiment 2 is identical to experiment 1.

In experiment 2, individual movements had a 120-mm center-to-center amplitude and were time constrained between 800 and 1,300 ms (matching the short-amplitude, mid-duration reaches of experiment 1, Fig 1F), for both individual reference movements and the test movements. The total duration of each action, starting from the time the subject left the fixation point to the end of the subtrial, was maintained constant over all subtrials by adding an additional waiting time when holding the target, resulting in a total subtrial duration of 4,000 ms. Contrary to experiment 1, both reference movements and the test movement were in the same direction in each trial. Four levels of reference movement force were probed (0, 3, 6, and 9 N), while the forces for the test movements were determined using the same staircase procedure as in experiment 1. Therefore, there was 1 staircase pair for each of the 8 conditions (4 reference movement force levels times 2 movement directions).

### Movement and choice analyses

Data processing and statistical analysis were carried out using Matlab and the gramm [34] toolbox for plotting.

In experiment 1, averaged staircase inversion points were analyzed using LMEs (fitlme function in Matlab). For each session, we constructed mixed-effect models fitting the average force at staircase inversion points depending on the varied parameter of the session (duration or amplitude: low, high), reference movement direction (relative to subject handedness: inward, outward), and reference movement force (low, high). All these independent variables were treated as categorical variables. The mixed-effect model included separate random intercepts and random slopes for movement duration and for amplitude across subjects. Main effect sizes were extracted from models without interaction terms. Interactions were tested in separate models and their significance was evaluated by model comparison.

Choice data from experiment 2 were modeled using Stan [35], a probabilistic programming language, through its Matlab interface. We used Stan to perform Bayesian inference, using its default implementation of a Markov chain Monte Carlo sampler (NUTS). We fitted a probit hierarchical model, in which the choice of the reference movement in each trial is modeled as a Bernoulli distribution in which the associated probability *P*(*R*|*F*_*T*_,*F*_*R*_) is a function of the difference in utility between the test and the reference movement, and the utility for each movement depends on the corresponding movement force. Variations of the model will differ in the way utility is expressed as a function of force-dependent effort.

Therefore, for subject *i*:

$$P(R|F_{T},F_{R}{)}_{i}=\Phi \left(\frac{U(2E_{i}(F_{R}))–U(E_{i}(F_{T}))}{\gamma_{i}}\right)$$ (1)

where *Φ* is the cumulative density function (CDF) of the standard normal distribution (probit link), *E*_*i*_(*F*) is the effort of a movement executed against the force *F* for subject *i*, and *U* is the utility as a function of force-dependent effort. The factor 2 reflects our assumption that repeating a movement twice should double the effort compared to a single movement and thus imposes the constraint *E*(*F*_*Teq*_) = 2*E*(*F*_*R*_). Effort itself in all variations of the model was modeled as power-law function of force:

$$E_{i}(F)=F^{\alpha_{i}}+\beta_{i}$$ (2)

The constraint *E*(*F*_*Teq*_) = 2*E*(*F*_*R*_), applied on Eq 2, yields the following equation for the equivalent force curve:

$$F_{Teq}=\sqrt[{\alpha_{i}}]{2{F_{R}}^{\alpha_{i}}+\beta_{i}}$$ (3)

The force exponent *α*_*i*_, the effort offset *β*_*i*_, and the effort sensitivity *γ*_*i*_ for each subject were drawn from normal distributions *α*_*i*_ ∼ *N*(*μ*_*α*_,*σ*_*α*_), *β*_*i*_ ∼ *N*(*μ*_*β*_,*σ*_*β*_), *γ*_*i*_ ∼ *N*(*μ*_*γ*_,*σ*_*γ*_). The resulting parameters of these normal distributions characterize the population-level distributions for *α*, *β*, and *γ*. Bayesian inference requires providing prior distributions for these parameters, which were chosen wide to not constrain the model: *μ*_*α*_ ∼ *N*(1,10), *μ*_*β*_ ∼ *N*(0,100). The parameters *μ*_*γ*_,*σ*_*α*_,*σ*_*β*_,*σ*_*γ*_ were positive scale parameters and their priors each followed the same half-Cauchy distribution [36] with parameters (location = 0; scale = 20). Posterior distributions were sampled using 4 Markov chains with 1,000 samples each (after a warmup of 1,000 samples).

To test our model against alternative hypotheses, we varied the function of the choice probability (Eq 1). We then compared the individual model fits using the WAIC [19], an approximation of cross-validation.

In our first model, utility is the negative logarithm of effort and choice probability thus depends on the difference between effort logarithms:

U(E)=−log⁡E (4)

As second model, we used a simpler model for the choice probability that is based on the difference of effort values and not the log-ratio (difference of their logarithms):

U(E)=−E (5)

Third, we tested the hypothesis of hyperbolic effort discounting with a model in which effort is on the denominator in each utility term (inverse effort):

$$U(E)=\frac{1}{E}$$ (6)

Finally, in a fourth model we modified the hyperbolic model to use logarithmic effort:

$$U(E)=\frac{1}{\log E}$$ (7)

## Supporting information

S1 Fig Successful constraining of movement parameters in Experiment 1. A. Trajectories along the x-axis in amplitude and duration sessions for subject LM between movement onset (t = 0) minus 100 ms and movement offset plus 400 ms. Colors correspond to amplitudes in the amplitude session panels (left), and to the duration in the duration session panels (right; colors correspond to the colors of the visual cue used in the task). Vertical dashed lines indicate the limits of the duration ranges. Inset: estimated positional oscillations due to the force oscillations. Dashed vertical lines indicate the peaks of the force oscillations. Black curve represents the oscillation average (160mm rightward movements, 6N force). B. Corresponding force profiles for subject LM, separated by condition and session. Lightness represents the different force levels. Inset: estimated force oscillations (same conventions and movements as in inset A). Data underlying this figure can be found at https://doi.org/10.6084/m9.figshare.4873055.v1.
(PNG)

S2 Fig Subjects’ effort estimates are performance-independent. A. Proportion of subject’s choices correctly explained by performance and force predictors in the amplitude session of Experiment 1, separated by test movement amplitude. With the “subtrial performance” predictor, the predicted choice for each trial corresponds to the subtrial which was failed the least amount of times in the trial (limited to trials where subtrials were failed a different amount of times). With the “average performance” predictor, the predicted choice for each trial corresponds to the alternative showing the best average performance across the whole session. With the “force” predictor, the predicted choice depends on the value of the test movement’s force relative to an optimal force threshold. Error bars correspond to bootstrapped 95% CI (from subject-level averages). The dashed horizontal lines correspond to the chance prediction levels for the different predictors. P-values indicate the results of generalized linear mixed-effect models (see Methods in main text). B. Proportion of subject’s choices correctly explained by performance and force predictors in the duration session of Experiment 1, separated by test movement duration. Same conventions as in A apply. B. Grand mean of subjects' performances across conditions of the amplitude session of Experiment 1, separated by movement amplitude (color) and resistive force (abscissa). Per-subject averages were computed over all subtrials. The shaded areas represent bootstrapped 95% confidence intervals (CI) of the grand mean. C. Grand mean of subjects' performances across conditions of the duration session of Experiment 1, separated by movement duration (color) and resistive force (abscissa). Other conventions are the same as in (B). D. Per-subject inter-quartile ranges of the test movement performance rates across test force levels, separated by movement amplitudes in the amplitude session (one point per subject/amplitude). E. Per-subject inter-quartile ranges of the performance rates across test force levels separated by movement duration in the duration session (one point per subject/duration). Data underlying this figure can be found at https://doi.org/10.6084/m9.figshare.4873055.v1.
(PNG)

S3 Fig Physical arm model for computing the torques developed by the subjects during Experiment 1. A. Assumptions of the model with respect to the performed task. We assume that the elbow E is coplanar with the shoulder S and the positions taken by the hand H. The model assumes that this plane is horizontal and thus ignores gravity. B. Details of the two-link arm model (the wrist W is not articulated) C. Average internal torques at the shoulder joint generated to compensate arm inertia for rightward movements against a force of 6N in the duration session (all subjects) D. Average internal torques at the shoulder joint generated to compensate the applied external force in the same trials. E. Estimated total torques applied by the subjects at the shoulder joint in the same trials (corresponds to the sum of torques C + D). Data underlying this figure can be found at https://doi.org/10.6084/m9.figshare.4873055.v1.
(PNG)

S4 Fig Rotational work and impulse for Experiment 1 taking into account a physical arm model, represented as in Fig 2 of the main manuscript. The plotted rotational impulse and work correspond to the sum of the corresponding values for the shoulder and elbow joint. Data underlying this figure can be found at https://doi.org/10.6084/m9.figshare.4873055.v1.
(PNG)

S1 Table Model parameters, adapted from Dempster (1955). Segment masses are expressed relative to body mass M. Moments of inertia are expressed using radii of gyration.
(XLSX)

S1 Text Experimental constraints on movement parameters. (DOCX)

S2 Text Subjects’ effort estimates are performance-independent. (DOCX)

S3 Text Applied manipulator force dominates movement torques. (DOCX)

## Abbreviations

2-AFC: 2-alternative forced choice
LME: linear mixed-effect model
MVC: maximum voluntary contraction
WAIC: Watanabe–Akaike Information Criterion
