# Supplementary figures and images for: What makes a reach movement effortful? Physical effort discounting supports common minimization principles in decision making and motor control
Source: PLoS Biol. 2017 Jun 6;15(6):e2001323. doi: 10.1371/journal.pbio.2001323 (PMC5460791; doi:10.1371/journal.pbio.2001323)

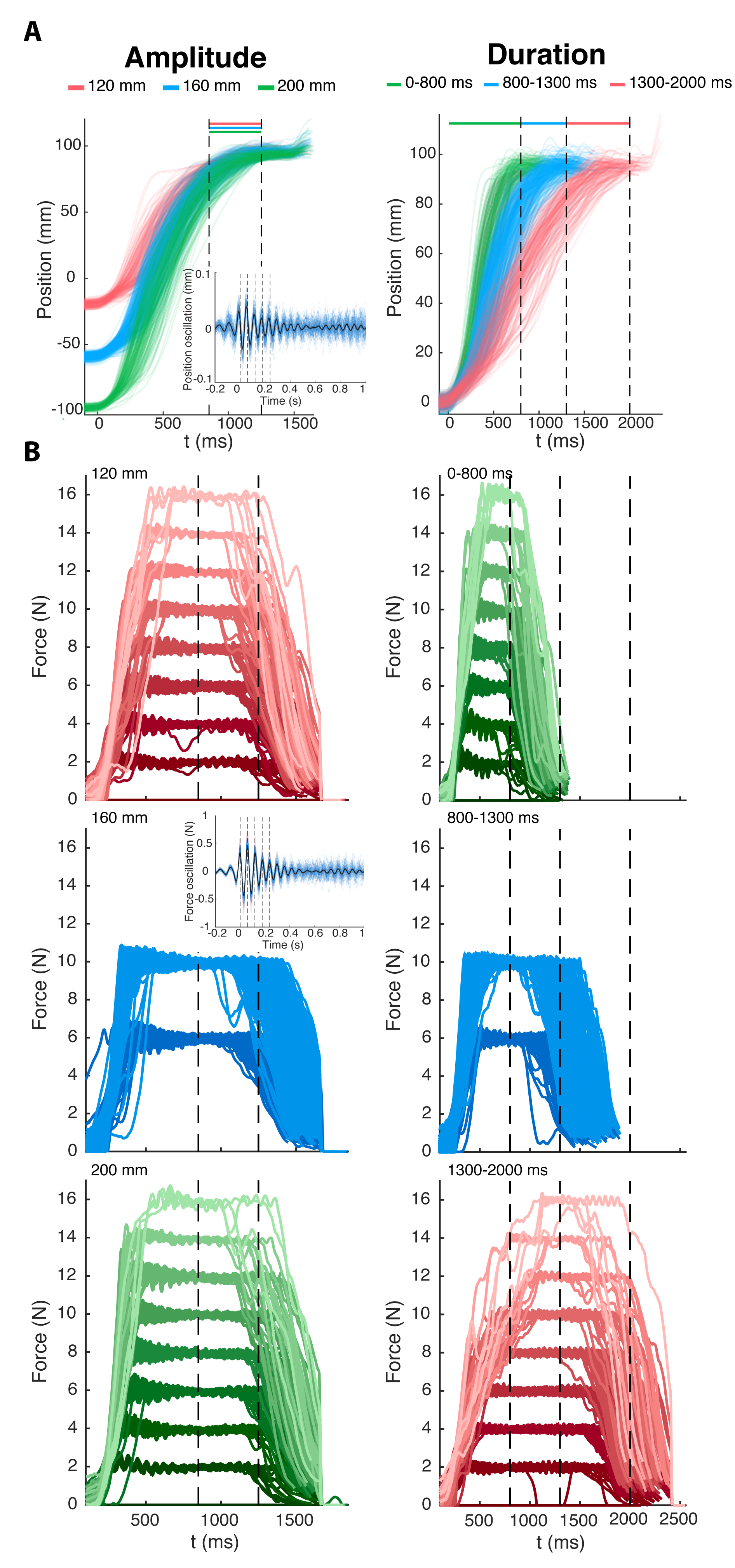

Supplement: S1 Fig — A. Trajectories along the x-axis in amplitude and duration sessions for subject LM between movement onset (t = 0) minus 100 ms and movement offset plus 400 ms. Colors correspond to amplitudes in the amplitude session panels (left), and to the duration in the duration session panels (right; colors correspond to the colors of the visual cue used in the task). Vertical dashed lines indicate the limits of the duration ranges. Inset: estimated positional oscillations due to the force oscillations. Dashed vertical lines indicate the peaks of the force oscillations. Black curve represents the oscillation average (160mm rightward movements, 6N force). B. Corresponding force profiles for subject LM, separated by condition and session. Lightness represents the different force levels. Inset: estimated force oscillations (same conventions and movements as in inset A). Data underlying this figure can be found at https://doi.org/10.6084/m9.figshare.4873055.v1. (PNG) [file pbio.2001323.s001.png]

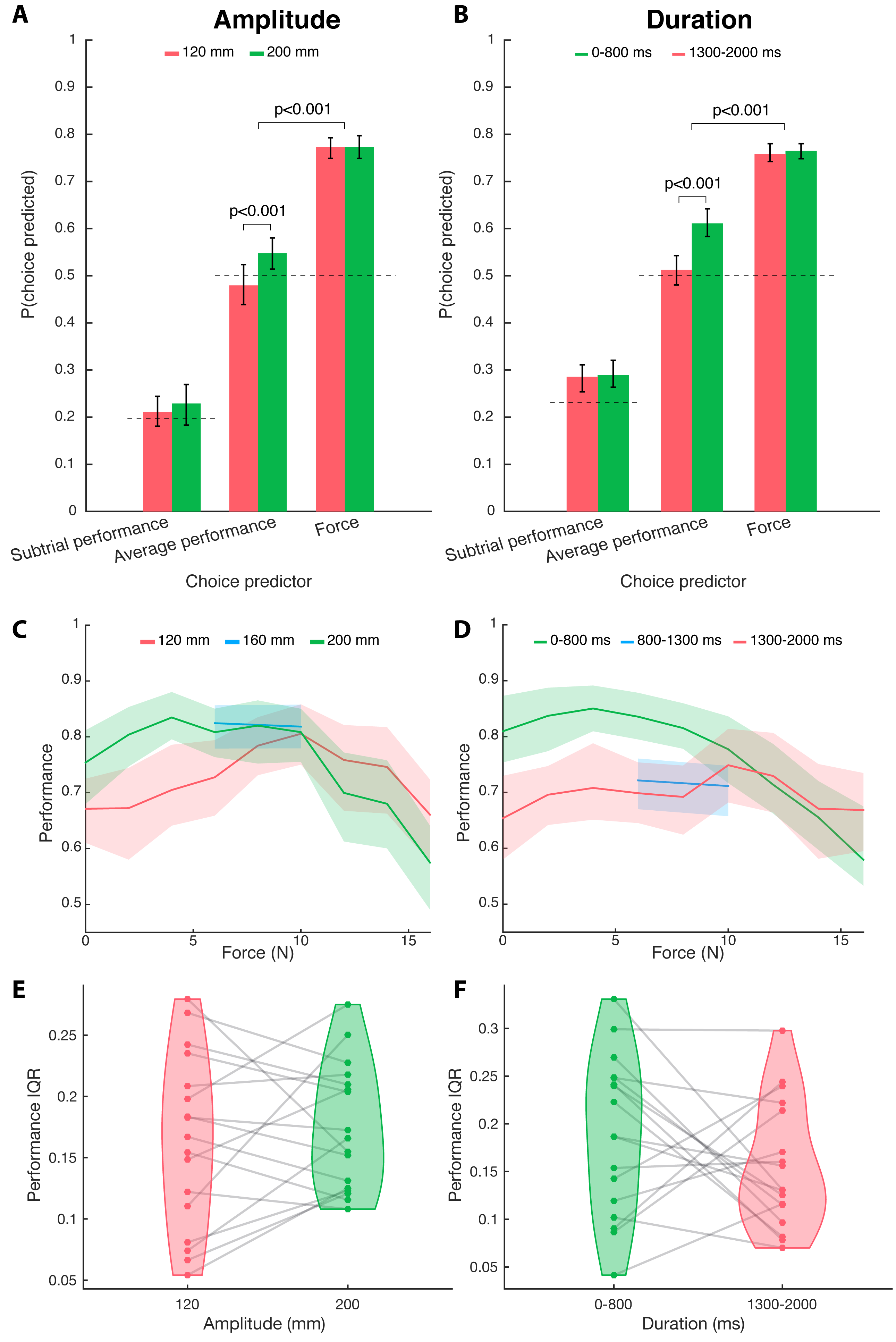

Supplement: S2 Fig — A. Proportion of subject’s choices correctly explained by performance and force predictors in the amplitude session of Experiment 1, separated by test movement amplitude. With the “subtrial performance” predictor, the predicted choice for each trial corresponds to the subtrial which was failed the least amount of times in the trial (limited to trials where subtrials were failed a different amount of times). With the “average performance” predictor, the predicted choice for each trial corresponds to the alternative showing the best average performance across the whole session. With the “force” predictor, the predicted choice depends on the value of the test movement’s force relative to an optimal force threshold. Error bars correspond to bootstrapped 95% CI (from subject-level averages). The dashed horizontal lines correspond to the chance prediction levels for the different predictors. P-values indicate the results of generalized linear mixed-effect models (see Methods in main text). B. Proportion of subject’s choices correctly explained by performance and force predictors in the duration session of Experiment 1, separated by test movement duration. Same conventions as in A apply. B. Grand mean of subjects' performances across conditions of the amplitude session of Experiment 1, separated by movement amplitude (color) and resistive force (abscissa). Per-subject averages were computed over all subtrials. The shaded areas represent bootstrapped 95% confidence intervals (CI) of the grand mean. C. Grand mean of subjects' performances across conditions of the duration session of Experiment 1, separated by movement duration (color) and resistive force (abscissa). Other conventions are the same as in (B). D. Per-subject inter-quartile ranges of the test movement performance rates across test force levels, separated by movement amplitudes in the amplitude session (one point per subject/amplitude). E. Per-subject inter-quartile ranges of the performance rates across test for [file pbio.2001323.s002.png]

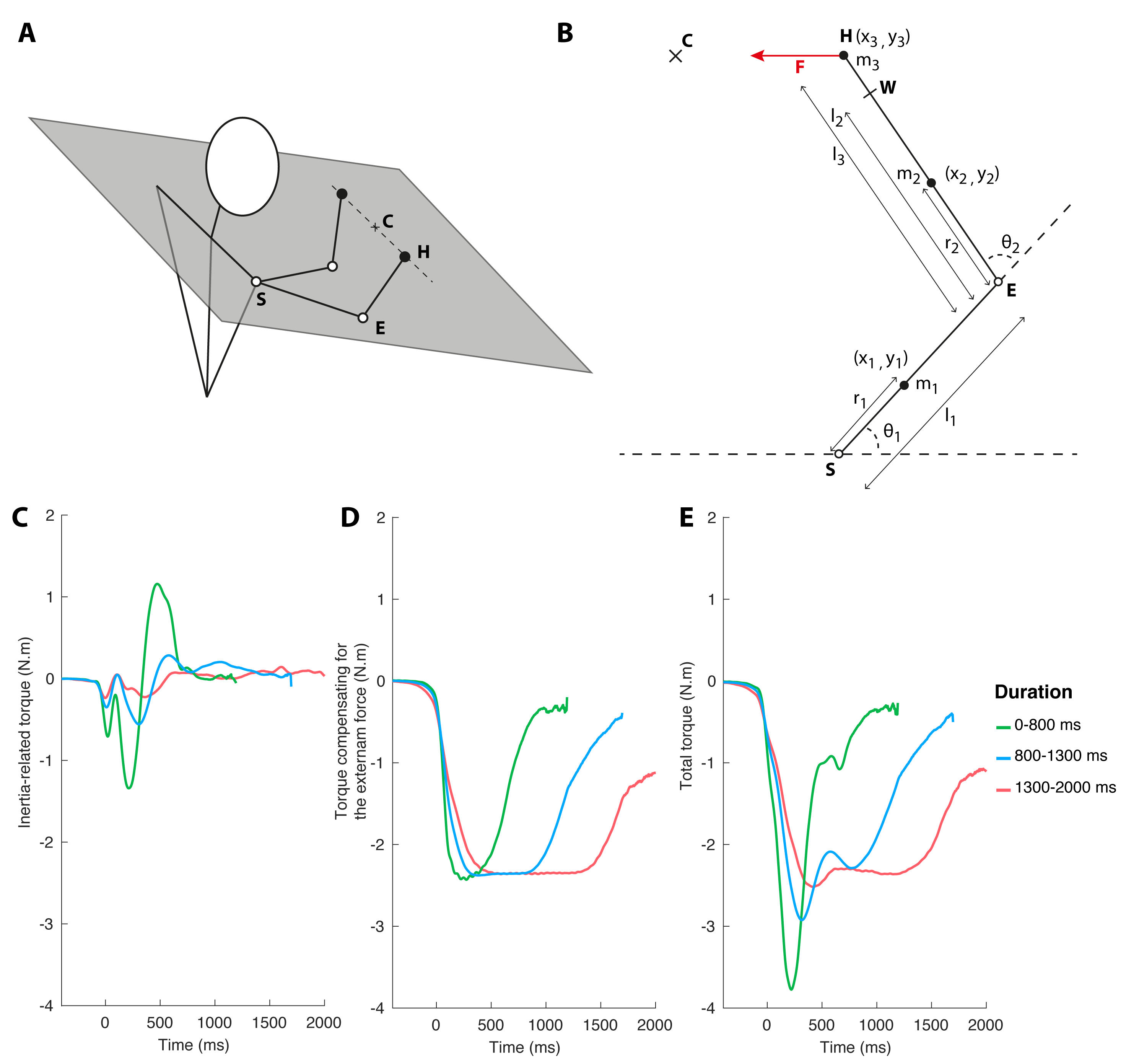

Supplement: S3 Fig — A. Assumptions of the model with respect to the performed task. We assume that the elbow E is coplanar with the shoulder S and the positions taken by the hand H. The model assumes that this plane is horizontal and thus ignores gravity. B. Details of the two-link arm model (the wrist W is not articulated) C. Average internal torques at the shoulder joint generated to compensate arm inertia for rightward movements against a force of 6N in the duration session (all subjects) D. Average internal torques at the shoulder joint generated to compensate the applied external force in the same trials. E. Estimated total torques applied by the subjects at the shoulder joint in the same trials (corresponds to the sum of torques C + D). Data underlying this figure can be found at https://doi.org/10.6084/m9.figshare.4873055.v1. (PNG) [file pbio.2001323.s003.png]

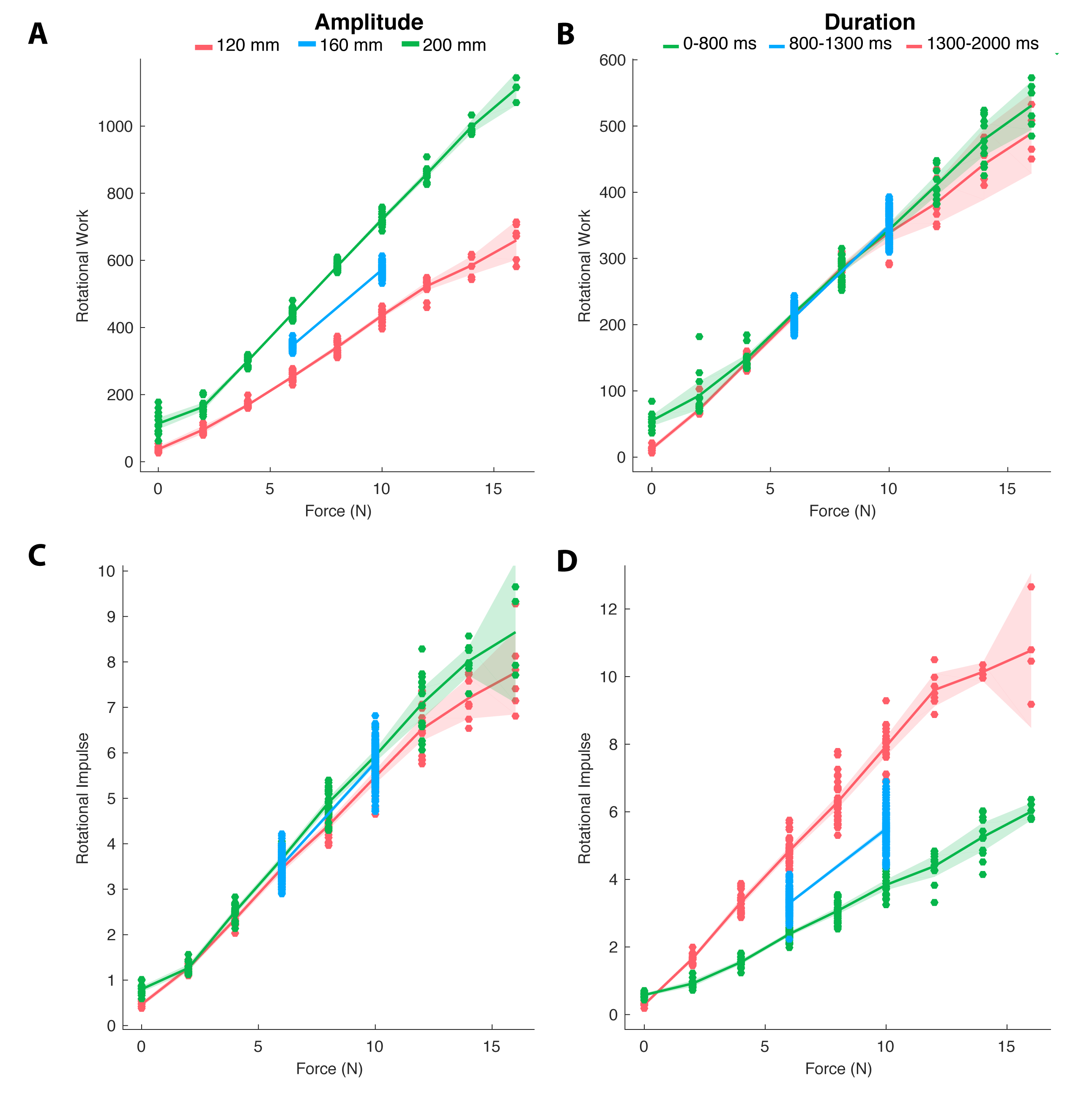

Supplement: S4 Fig — The plotted rotational impulse and work correspond to the sum of the corresponding values for the shoulder and elbow joint. Data underlying this figure can be found at https://doi.org/10.6084/m9.figshare.4873055.v1. (PNG) [file pbio.2001323.s004.png]
