# Supplementary material for: What makes a reach movement effortful? Physical effort discounting supports common minimization principles in decision making and motor control
Source: PLoS Biol. 2017 Jun 6;15(6):e2001323. doi: 10.1371/journal.pbio.2001323 (PMC5460791; doi:10.1371/journal.pbio.2001323)
Supplement: S1 Text — (DOCX) [file pbio.2001323.s006.docx]

Experimental constraints on movement parameters

We needed to ensure that with our haptic interface and the subject instructions we achieved proper control of all task-relevant movement parameters. Reach amplitude was determined by the spatial arrangement of starting point and reach target, and their acquisition controlled on-line by the task control software via the precise position read-out of the robot. Similarly, compliance with time constraints was supervised on-line via the precise timer in the control software. Force, instead, was part of the robotic control algorithm, implemented via a virtual mass and spring. Here we want to confirm that subject indeed conducted smooth movements with constant (except for the on- and off-tapering) force at the desired force levels.

First, subjects performed stereotypical reaching movements within the enforced duration and amplitude boundaries. Fig S1A depicts the successful trajectories for a representative subject for all trials of both sessions. The influence of the requested movement parameters on the subjects’ performance will be presented below.

Second, the force profiles show a correct separation between the different force levels (see Fig S1B for an example subject). The tapering of the forces at the beginning of the movement was due to the way the constant force levels were computed, and corresponded to the loading of a virtual spring (see Methods). Small oscillations that can be observed on the force profiles had the same cause (extracted oscillations in inset of Fig S1B). These small force oscillations did not affect the outcome of the experiments: they were not reported by the subjects in our post-experiment questionnaires, did not affect movement performance or force discriminability, and caused only negligible oscillations of the robot handle (deviations of less than 50 µm from the trajectory, inset in Fig S1A). We generated the oscillations estimates by aligning the signals to the first force oscillation peak and plotting the difference between two low-passed versions of the signals (one with a 12Hz cutoff to remove the 17Hz oscillations, and the other with a 25Hz cutoff to keep them – 20^th^ order Butterworth filters). The tapering of the forces at the end of the movement was a combination of the unloading of the virtual spring and the tapering of an envelope function applied to the force output. While the tapering appears to last for a larger proportion of the movement in short-duration trials, the shape of the tapering did not affect adversely the movement-related variables work and impulse which we intended to constrain: both, work and impulse turned out to be linear functions of force level as intended (see Fig 2 in main document).
