# Supplementary material for: What makes a reach movement effortful? Physical effort discounting supports common minimization principles in decision making and motor control
Source: PLoS Biol. 2017 Jun 6;15(6):e2001323. doi: 10.1371/journal.pbio.2001323 (PMC5460791; doi:10.1371/journal.pbio.2001323)
Supplement: S2 Text — (DOCX) [file pbio.2001323.s007.docx]

Subjects’ effort estimates are performance-independent

The utility of an action alternative depends on the expected success probability associated with performing the required movement. In both experiments, subjects were instructed to base their choice exclusively on experienced physical effort during the sampling subtrials, but despite this instruction their choices could still be biased by the expected performance. Therefore, we needed to rule out that subjects’ choices and hence our conclusions on effort were confounded by performance differences.

In Experiment 1, the different conditions of the task corresponded to different constraints on the movements the subjects had to perform. It was possible for the subjects to fail to meet these imposed constraints, and subtrials were repeated until successfully completed. We can thus test if the subject’s choice in a trial was biased when one subtrial was failed and repeated more often than the other. Average movement success rate across subjects was 78% in the amplitude session, and 72% in the duration session. Among trials in which one of the sampling subtrials was repeated more times than the other (39 % and 46 % of the trials in the amplitude and duration session, respectively), the subjects showed an above-chance tendency to choose the least repeated option in both the amplitude session (bootstrapped 95% CI: 53 – 60%; chance level 50%) and the duration session (bootstrapped 95 % CI: 60 – 67%). When reported over all trials (Fig S2A and S2B), the trials in which choice followed the sampling subtrial which required less repetitions amount to 22 % in the amplitude session and 29 % in the duration session. Therefore, even if there was a direct biasing effect on choices by the preceding subtrial performance, it is limited to a fraction of trials, and differs between sessions, suggesting that performance, if at all, it is not a major confounding explanation for our results. In the following, we will provide a more stringent control and argue why we think that performance expectation actually did *not* determine choice.

In trials for which both sampling subtrials were not repeated or repeated equally often, subjects’ choices could potentially even be influenced by movement performance beyond the immediately preceding subtrials. Therefore, as the most stringent control for the question of whether performance affected choices, we analyzed the percentage of choices explained by the average subjects’ performances over the whole session. This corresponds to the “average performance” category of Fig S2A and S2B, in which for each trial the hypothesized subjects' choice corresponds to the option with the highest average performance (performance averages were computed within subject, movement direction, duration, amplitude, and force). We compared these performance-based choice predictions to force-based choice predictions, which correspond to our working hypothesis: for each staircase pair, we computed a test movement force threshold that explains the subject’s choices best. This approach for computing force thresholds gives results similar to the average staircase inversions presented in the main text. On average, the performance-based choice prediction performed significantly worse than the force-based prediction, and did not even exceed chance level in the amplitude session (performance-based prediction accuracy CI: 49 – 53% and 54 – 58% in the amplitude and duration sessions, respectively; force-based prediction accuracy CI: 76 – 79% and 74 – 78%, p<0.001 generalized LME across both sessions).

To further understand the potential effect of performance on choice, and compare it to the effect of force on performance, we separated the predictions by type of test movement (low/high amplitude/duration). This separation shows that performance-based choices are only present for rapid test movements, namely movements toward a distant target in the amplitude session and movements of short duration in the duration session (Fig S2A and S2B). Indeed, only rapid test movements led to above-chance performance-based choice predictions, and showed significantly higher prediction accuracy than trials with slow test movements (p<0.001 for separate generalized LMEs in amplitude and duration session). This difference parallels observations made on the effect of force on performance (Fig S2C and S2D). There, only conditions with rapid movements showed a significant linear drop of performance with force (amplitude session, distant target: slope= -0.065, p<0.001; duration session, small duration: slope= -0.091, p<0.001 respectively, separate generalized linear mixed-effect models with force as a continuous parameter and subject as random parameters, Bonferroni-corrected $\alpha$ level for 6 tests: 0.0083). While this performance dependence on force in the subgroup of fast movement conditions existed as linear trends across the subject population, they do not indicate that within-subject performance varied less across force levels for slow movements compared to fast movements. Indeed, both movement regimes showed similar variability in their performances across force levels (Wilcoxon sign-rank test comparing performance inter-quartile ranges computed within subjects across movement speeds, p=0.43 and p=0.21 in amplitude and duration session respectively, Fig S2E and S2F). Both slow and rapid test movements thus presented enough variability in their performances across forces to potentially support performance-based choices, yet only rapid test movements (in which force consistently influenced performance) did so. In summary, we observed apparent performance-based choices only in conditions where performance fluctuations were linearly correlated with force. But since overall choice was much better explained by force than by performance, one has to assume that the explanatory power of performance for choice is a by-product of the anti-correlation between force and performance, rather than an independent cause of choice. In other words, there is likely no direct causal effect of performance on choices, but some choices appear to be performance-based in conditions where force effects and performance are anti-correlated.

Results from experiment 2 also argue against a performance-based choice behavior of the subjects. In experiment 2, we verified that in the reference subtrials, the error rates of the two constituting movements were the same as the error rates of the equivalent single movements in test subtrials. For this, the overall success rate for the reference subtrials (i.e. the overall success rate over both movements) should be equal to the square of the success rate for single movements (assuming statistical independence of performance in the two repeated movements). This was the case in the range of forces over which test and reference actions overlapped and hence allowed us to verify this prediction (respective performances 52% and 72% in the 0-10 N range, with ${0.72}^{2}\approx0.52$).

For the same reasons as in Experiment 1, the observed choice behavior in Experiment 2 was better explained by force-based choices rather than performance-based choices. Indeed, we also observed a small portion of trials where choices could appear performance-based (performance-based prediction accuracy 95% CI: 54 – 59%, chance level 50%), while force-based choice predictions were more accurate (force-based prediction accuracy 95% CI: 73 – 77%; p<0.001 when compared to performance-based prediction with generalized LME). Moreover, as in the rapid movements conditions of Experiment 1, we observed that performance dropped significantly with increased force in Experiment 2 (generalized LME, p<0.001, generalized LME in test sampling trials), which again gives rise to apparent performance-based choice without a causal link between performance and choice.
