# Supplementary material for: What makes a reach movement effortful? Physical effort discounting supports common minimization principles in decision making and motor control
Source: PLoS Biol. 2017 Jun 6;15(6):e2001323. doi: 10.1371/journal.pbio.2001323 (PMC5460791; doi:10.1371/journal.pbio.2001323)
Supplement: S3 Text — (DOCX) [file pbio.2001323.s008.docx]

**Applied manipulator force dominates movement torques**

A potential concern in our experiment is the difference between the forces imposed by the manipulator on the hand (external forces) and the internal torques the subjects had to produce during the movements to counteract both the imposed external force and the inertia of their arm. To test for the quantitative influence of the inertia, we built a simple dynamical model of the arm in order to estimate torques compensating for both the inertia of the arm and for the external forces. Results from this model confirm that taking into account inertia-related torques does not affect our conclusions and our interpretations.

In order to keep the arm modeling tractable, we had to make simplifications. First, we considered that the arm is moving in a plane, i.e. that the elbow joint is coplanar with the shoulder joint and the hand trajectory (Fig S3A). The shoulder position was reasonably well fixed and hand trajectories were horizontal in our experiment. Yet, it is likely that during the experiment, the subjects’ elbow lied below the shoulder-hand plane, as this is a more natural position. Arm movements generated from this natural posture, where the upper arm rotates about the axis of the humerus, should cause smaller rotational kinetic energies than the posture modeled here. With our model, we thus obtain a conservative estimate of the inertia-related torques in the sense that we rather over- than underestimate them. Second, since the hand movements were horizontal, we ignored gravity. Third, we modeled the arm as having two segments, and ignored the wrist joint (Fig S3B). These simplifications allowed us to obtain unique solutions for the shoulder and elbow angles as a function of hand position.

We modeled the physics of the arm using the Lagrange formulation, with shoulder and elbow angles (**Θ**_1_ and **Θ**_2_) as generalized coordinates. In these coordinates, we thus expressed linear kinetic energy terms for the upper arm (mass m_1_ at (x_1_,y_1_)), the forearm (mass m_2_ at (x_2,_y_2_) and the hand/robot (mass m_3_ at (x_3_,y_3_)), as well as rotational kinetic energy terms for the upper arm (moment of inertia i_1_), the forearm (moment of inertia i_2_) and the hand/robot (moment of inertia corresponding to a point mass). The values used for the model are detailed in Table S1.

Using hand position data from subjects (extracted between movement start minus 400ms and movement end plus 400ms), we computed shoulder and elbow angles as well as their first and second time derivatives using inverse kinematics. Using these values and the Euler-Lagrange equation including the terms described above, we obtained generalized forces for each joint, which correspond to the sum of the torques generated by the subjects and the generalized force imposed by the robot. The latter can be expressed as a function of the imposed force and the joint angles. We were thus able to generate estimates for the torques generated by the subjects for each joint (Fig S3 CDE).

From the estimated torques, we computed rotational work and impulse estimates in order to generate the results corresponding to Fig 2 in the main manuscript, but now taking into account the physics of the arm (Fig S4; impulse $J_{i}=\int_{t_{start}}^{t_{stop}}\left| \tau_{i} \right|dt$ and work $W_{i}=\int_{t_{start}}^{t_{end}}\left| \tau_{i}\frac{d\theta_{i}}{dt} \right|dt$). This figure is highly similar to Fig 2 in the main manuscript, and the double-dissociation between work and impulse we had intended to create with the amplitude and duration sessions is fully conserved (i.e. there are no impulse differences in the amplitude session, and no work differences in the duration session). Indeed, the speed-independent force profiles we imposed on the subjects had a purely additive effect on the torques, and dominated the work and impulse the subjects had to generate to simply counter inertia (Figure S4 C-E). For example, in the duration session, the impulse (Fig S4 D) is larger for fast movements than slow movements only in trials against no force (0N).
